# Examination of the interactions between the HTLV-1-encoded Tax oncoprotein and the cellular coactivators CBP/p300

**DOI:** 10.1186/1742-4690-11-S1-P130

**Published:** 2014-01-07

**Authors:** Alisha D Howard, Jennifer K Nyborg

**Affiliations:** 1 Department of Biochemistry and Molecular Biology, Colorado State University, Fort Collins, CO, USA

## 

Human T-cell Leukemia virus type-1 (HTLV-1) causes several diseases including aggressive Adult T-cell Leukemia (ATL). A major player in the malignant transformation of infected cells is expression of Tax, the virally-encoded oncoprotein. Tax is a potent activator of HTLV-1 transcription. To activate viral transcription, Tax, together with the cellular protein CREB, bind to the viral CRE enhancer elements located in the HTLV-1 promoter. Together, these activators recruit the homologous coactivator proteins CBP to initiate strong transcription of the provirus. Both CBP/p300 are involved in multiple cellular pathways, and deregulation of these proteins is associated with numerous pathologies. Despite extensive work on Tax-stimulated recruitment of CBP/p300 to the HTLV-1 promoter, primarily via their KIX domain, potential interactions between Tax and other domains of CBP/p300 have not been fully investigated. Recently, we have found that Tax interacts with several additional domains of the coactivators, however the significance of these interactions remains incompletely understood. We hypothesize that Tax interaction with various domains of CBP/p300 stimulates the potency of the coactivators and enhanced transcription. Our studies utilize protein-protein and protein-DNA interaction assays using individual domains of the coactivators, together with mutations and deletions that specifically target the Tax-CBP/p300 interface. Competition and activity assays enable us to functionally and biophysically characterize Tax-coactivator interactions. These studies have implications for both viral and cellular mechanisms of gene expression, and expand our understanding of the role of Tax and the cellular coactivators in HTLV-1 transcription and replication.

